# Network based elucidation of drug response: from modulators to targets

**DOI:** 10.1186/1752-0509-7-139

**Published:** 2013-12-13

**Authors:** Francesco Iorio, Julio Saez-Rodriguez, Diego di Bernardo

**Affiliations:** 1 European Molecular Biology Laboratory - European Bioinformatics Institute, Wellcome Trust Genome Campus, Cambridge CB10 1SD, UK; 2 Telethon Institute of Genetics and Medicine, Naples, Italy; 3 Cancer Genome Project, Wellcome Trust Sanger Institute, Hinxton CB10 1SA, UK; 4 Deptartment of Electrical Engineering and Information Technology, University of Naples “Federico II”, Naples, Italy

**Keywords:** Network pharmacology, Drug mode of action, Drug repositioning

## Abstract

Network-based drug discovery aims at harnessing the power of networks to investigate the mechanism of action of existing drugs, or new molecules, in order to identify innovative therapeutic treatments. In this review, we describe some of the most recent advances in the field of network pharmacology, starting with approaches relying on computational models of transcriptional networks, then moving to protein and signaling network models and concluding with “drug networks”. These networks are derived from different sources of experimental data, or literature-based analysis, and provide a complementary view of drug mode of action. Molecular and drug networks are powerful integrated computational and experimental approaches that will likely speed up and improve the drug discovery process, once fully integrated into the academic and industrial drug discovery pipeline.

## Background

A network is a natural abstraction of a set of objects (nodes) and of the relationships (edges) occurring among them. Nodes and edges in a network may represent heterogenous kinds of relationships, according to the phenomenon being modelled.

Networks have been extensively used to represent regulatory and functional interactions among genes, proteins and metabolites, by mapping experimentally verified, or computationally predicted, interactions as edges between the corresponding nodes [1]. Large-scale genomic, transcriptomic and proteomic experimental data enable the identification of thousands of interactions in a relatively short time, even though their functional meaning is not immediately evident [1,2].

The added value of representing interactions among molecular species as a network stems from the existence of well established theorems and algorithms to identify network level properties, which are not apparent when looking at single interactions [3,4].

Network-based drug discovery and systems pharmacology aim at harnessing the power of networks to investigate the impact of small molecules on molecular networks in order to elucidate their mechanism of action and to identify innovative therapeutic treatments [5]. These innovative methodologies can be used to discover: (i) on-target effects, i.e. the intended physical drug-substrate interactions, thus helping in the drug discovery process during lead optimisation (ii) off-target effects, i.e. unforeseen direct physical drug-substrate interactions, and (iii) indirect effects, due to signal propagation after the direct interaction between a drug and its substrates, thus helping in the identification of novel therapeutic opportunities for drug repositioning.

Here, we will review some of the recent advances in the field of network pharmacology, starting with approaches relying on transcriptional networks, then moving to protein and signaling networks and concluding with “drug networks”. We will show examples of applications of these methodologies both in drug discovery and in drug repositioning.

## Identifying drug mode of action: Transcriptional networks

**Figure 1:**
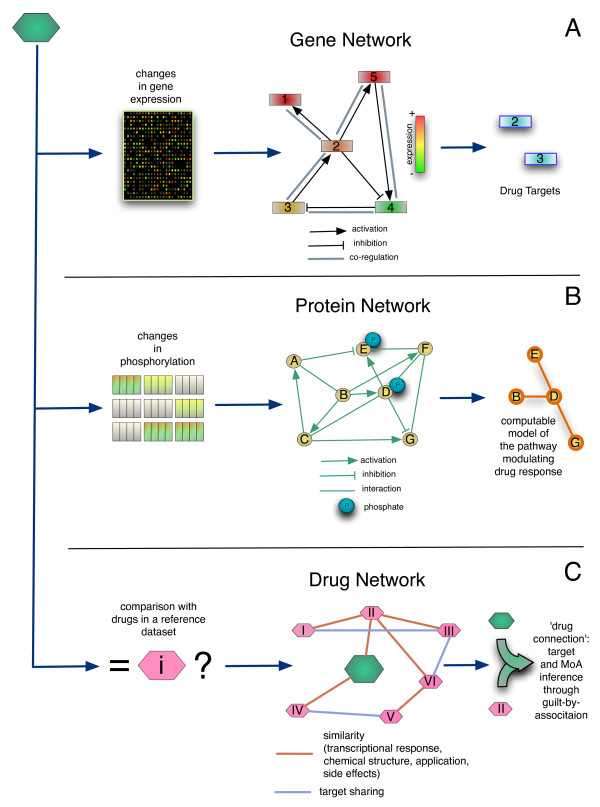
**Network models can be used in combination with experimental data to dissect drug mode of action and for drug repositioning.(A)** In transcriptional networks nodes are individual genes and edges represent pair-wise functional or regulatory interactions. These networks can be “reverse-engineered” from gene expression profiles (GEPs) with different computational methods or derived from literature. Transcription network models can be used to filter for GEPs following drug treatment in order to infer the primary targets causing the observed ranscriptional changes. **(B)** Protein interaction networks can be used to model signaling pathways, where edges imply phosphsorylation/de-phosphorelation events. Signaling network models can be inferred from phosphoproteomic data. These models can be used to simulate in-silico the drug effects on signal transduction. **(C)** Drug similarity networks describe similarities between drugs, such as similar transcriptional responses or similar adverse-reaction. Drug networks can be easily inferred from gene expression profiles following multiple drug treatments.

Transcriptional (or gene) networks can be broadly defined as a set of nodes representing genes and possibly non-coding RNAs, and a set of edges among genes interacting at the regulatory or functional level (Figure 1). These connections are not necessarily physical interactions, as in the case of protein networks, but can also represent indirect statistical dependencies between genes or ncRNAs [6]. Usually, edges are inferred (“reverse-engineered”) from Gene Expression Profiles (GEPs) through computational analysis. Gene expression data from microarrays are typically used for this purpose, but it is likely that Next Generation Sequencing techniques will soon replace them. A gene network can also be compiled using literature-based approaches, without directly using any experimental data.

The gene network paradigm can be used to represent any one gene in the context of a molecular network that defines the cell behaviour in physiological and pathological conditions [5]. Once a gene network model for a specific cell type or tissue is available, it can be used to “filter” the downstream response of a biological system to a small molecule (or a disease) to identify or confirm its direct molecular targets, as shown in Figure 1. Indeed, changes in the direct targets’ activity propagate to other genes through the network, and cause the observed phenotypic response. Thus, gene networks allow to investigate molecular targets of existing drugs, for drug repositiong, as well as to optimise libraries of lead compounds for drug development.

Here, we will review the recent progress made in using gene networks as a tool to elucidate the Mode of Action (MoA) of a compound, that is, the genes and pathways directly modulated by it.

### Inference of gene network and drug MoA from steady-state gene expression profiles

One of the first applications of a gene network reverse-engineering approach to identify compound Mode of Action relied on a technique named Network Inference by Regression (NIR) [7].

The authors applied the NIR algorithm in bacteria to reverse-engineer a gene network model consisting of nine genes of the DNA repair SOS pathway; they then used this network to identify direct targets of the chemotherapeutic agent Mitomycin C.

NIR uses multiple linear regression to infer a gene network model from RNA expression changes resulting from a set of **steady-state** transcriptional perturbations. Specifically, each gene in the SOS pathway was perturbed (i.e. over-expressed) and the transcriptional response of all the genes in the network measured. At the end of the inference procedure, the resulting network was represented by a set of linear differential equations. Each equation described the rate of accumulation of one gene in the network as a linear combination of its regulators, and possibly, of an external perturbation (e.g. a small molecule).

To identify the direct targets of Mitomycin C, the authors first measured RNA expression changes at steady-state resulting from treatment with the compound (i.e. at a single time point following drug administration). The activity of the compound was modeled as a set of unknown external perturbations acting on one, or few genes, in the network. By filtering the differentially expressed genes through the differential equation model of the gene network, the authors were thus able to identify which genes in the network were direct targets of Mitomycin C, and to distinguish them from indirect targets (Figure 1A).

This study represented an interesting proof-of-principle, but it was limited in that the inferred gene network was not genome-wide but included only a few selected genes; in addition, the method required each of the gene in the network to be perturbed, by either silencing its expression, or by inducing it, thus making the scaling up of the approach to the genome level very difficult.

In a subsequent study, an extension of the NIR approach, named Mode of action by Network Identification (MNI), was described [8,9]. MNI does away with the requirement of having to perturb each of the genes in the network, thus making the approach scalable to the genome level. In MNI, the network is modelled as a system of linear differential equations, as in NIR, and the inference of the network (i.e. the equation parameters) is performed on a compendium of GEPs; the number of GEPs needed for inference, differently from NIR, can be much smaller than the number of genes in the network, thus making the approach easily scalable. Once the network is inferred, it is used to “filter” a single GEP obtained by treating cells with a compound of interest, and to rank genes according to their probability of being the direct targets of the small molecule.

MNI was first applied to a compendium of genome-wide GEPs measured in yeast (S. cerevisiae) to identify the molecular targets of anti-fungal compounds [8]. Subsequently, MNI was applied to genome-wide GEPs measured from seven different human cancer cell lines to identify the androgen receptor gene as a genetic mediator of recurrent and metastatic prostate cancer [10].

In the cancer research field, the gene network framework has been applied successfully to identify dysregulated pathways due to oncogenic lesions [11]; a similar approach could be used to identify molecular targets of a small molecule. An algorithm named Interaction Dysregulation Enrichment Analysis (IDEA) has been recently proposed [11]. The method was applied to identify oncogenic lesions in lymphomas. IDEA starts with an “a priori” network model in B cells, assembled by merging different types of experimental and predicted interactions (i.e. protein-protein, co-regulation, etc.), it then uses two sets of GEPs, one comprising GEPs measured in normal B-cell populations and another set measured in B-cell populations from lymphoma patients. By computing differences in co-regulation between genes across the two datasets, using a Mutual Information measure, IDEA was able to identify dysregulated pathways in disease. Although, this method was not applied to identify targets of a small molecule, this in principle could be done. In this case, however, more than one GEP following drug treatment should be available, since Mutual Information requires more than one GEP to be computed. One could compare, for example, drug treated samples to untreated samples and predict pathways targeted by the compound.

### Inference of gene network and drug MoA from time-course gene expression profiles

The methods reviewed so far are all based on the availability of several GEPs (in the order of 10^2^ for genome-wide applications) measured at a single time point (steady-state) following several different perturbations. These GEPs are then used to infer a gene network model, which in turn is used to filter the drug-induced GEP measured at a single time-point following drug administration.

It is also possible, however, to use a single perturbation, i.e. treatment with the compound of interest, but at multiple time-points (i.e. a time-series) following the drug treatment. From this time-series data, it is then possible to identify the direct molecular targets of the compound and to distinguish them from indirect responses of downstream genes.

An algorithm named Time Series Network Analysis (TSNI), based on this approach, has been proposed by Bansal et al. [12] and applied to infer the targets of the antibiotic Norfloxacin in E. coli. TSNI is similar to NIR and MNI, since it uses a set of linear differential equations to describe the gene network model. Differently from NIR and MNI, however, time-series data are used to identify the equation parameters and detect the direct targets of the perturbation. In this study [12], the authors measured gene expression profiles of nine genes in the SOS pathway at six time-points following treatment of E. coli cells with the antibiotic Norfloxacin. They then applied TSNI to identify the direct mediator of Norfloxacin response among the nine genes.

TSNI was also applied to mammalian cells at the genome-wide level to identify the direct transcriptional targets of the p63 transcription factor in primary murine keratinocytes [13]. The authors measured time-course gene expression profiles at fourteen time-points following inducible activation of the transcription factor using microarrays. TSNI was then applied to the collected GEPs to identify the direct targets of the transcription factor. More recently, TSNI was applied to identify the transcriptional target of Id proteins following inducible deletion of *Id* genes in murine Neuron Stem Cells [14].

Other methods using time-course gene expression profiles have been developed to reconstruct gene regulatory networks [15,16] and to infer the direct transcriptional targets of a Transcription Factor (TF) [17,18]. Some of these methods make use of dynamic Bayesian networks and are based on hidden variables that can capture effects not directly detectable in a gene expression profiling experiment (i.e. genes that have not included in the microarray, levels of regulatory proteins, effects of mRNA and protein degradation) [15,16]. To model the effect of the TF on each of the genes and to distinguish direct gene targets from indirect targets of the TF, it is also possible to use simplified model of gene regulation, based on linear differential equations and Gaussian Processes [17,18]. Although the authors of this work did not mention their use to identify drug MoA, in principle these approaches may be applied in a similar fashion to TSNI, if the time-course GEPs are measured following treatment with a compound of interest.

### Literature-derived gene networks for identification of drug MoA

Literature derived gene and protein networks, obtained by manual curation based on published literature, are a popular way to interpret differentially expressed genes following drug treatment and to identify potential pathways and molecules targeted by the drug. Several tools have been developed to assemble and analyse literature derived biological networks [19]. Usually their interpretation is done by visual inspection, which although useful, cannot be considered as an objective criterion. Different methods have been proposed to solve this problem. Carro et al. applied an algorithm, named Master Regulator Analysis (MRA), which uses a glioblastoma-specific gene network to analyse a "mesenchymal" gene expression signature (MGES), consisting of genes differentially expressed in poor prognosis group of glioma patients in [20]. The algorithm computes the statistical significance of the overlap between the genes connected to each TF in the gene network and the MGES genes, and ranks all the TFs by their likelihood of being direct regulators of the genes in the signature. This algorithm may be used with a literature derived gene network and a list of differentially expressed genes following drug treatment to identify the likely mediators of the drug response.

Along the same lines, Kotelnikova *et al* presented an algorithm named SubNetwork Enrichment Analysis (SNEA), which uses a similar idea as the MRA algorithm described above: genes differentially expressed in muscle biopsies from Duchenne Muscular Dystrophy patients were mapped to a literature-curated gene network to find master regulators of the differentially expressed genes; a similar approach could be used to elucidate drug mode of action by using differentially expressed genes following drug treatment [21].

## Identifying drug mode of action: protein and signaling networks

Several drugs, such as chemotherapeutic agents, exert their action by affecting the activity of proteins part of the signal transduction machinery. Therefore, the study of signaling networks has potential to enhance our understanding of drug’s mode of action. Methods for analyzing protein signaling networks are significantly less mature than those for gene regulatory networks, both experimentally and computationally [22]. Nevertheless, some promising approaches have been proposed in the literature and progress is being made at a fast pace. In what follows, we will review some recent applications making use of protein networks to study in silico how drugs operate by perturbing signal transduction pathways (Figure 1B).

The main common feature among the different methods in the literature is the conversion of a protein network into a computational model able to replicate in silico the signaling network function, including its response to perturbations such as drug treatments. These models, in turn, allow mechanistic studies of drug response, with the aim of understanding how interactions between drugs and their targets affect other proteins and cellular components, and thereby cellular phenotype [22].

There are multiple approaches to construct a mathematical model of a signaling pathway with different level of details [23]; more detailed models require more data and knoweldge, thus limiting their scope. We will consider two variants: models that describe signal transduction on the basis of its underlaying biochemistry, which provide detailed mechanistic insight, and others that follow a coarser approach based on describing signaling networks as logic circuits, which provide less detail but cover larger networks.

### Biochemical models

The most common approach to model signal transduction consists of formalising the corresponding biochemical processes and derive from them a dynamic mathematical model (typically as set of differential equations). This feature makes them a natural frame to study drug mode of action, as they can accommodate detailed molecular mechanisms. Furthermore, they can include non-linear effects, allowing to study complex behaviours such as the synergistic combinations of drugs [24,25].

Biochemical models contain a large number of parameters (such as binding constants or total amount of proteins) that are often unknown. These parameters can be found from literature or by training the models on dedicated experimental dataset. Since phosphoproteomic data are relatively difficult to collect, when compared to gene expression data, biochemical models rarely cover more than a couple of pathways and a dozen of proteins [23].

Once a model is set up and the parameters determined, one can analyze it with various techniques. For example, sensitivity analysis provides information on the effect of changes in parameters (such as binding affinities) on the state of model variables (such as the phosphorylation of key protein). This technique can be used to find points of intervention to be targeted with drugs [26] (Figure 1B). Schoeberl *et al* built a model fo the Receptor Tyrosine Kinase Family ErbBB and their effect on the key oncogenic pathways MAPK and AKT [27]. Sensitivity analysis revealed a previously unappreciated phenomenon: changes in the amount of ErbB3 receptor affected the phosphorylation of AKT much more than changes in ErbB1 and ErbB2 (which had been the focus of drug development). Subsquently, the authors developed MM-121, a human antibody that binds specifically to ErbB3, blocking HRG1-b binding to ErbB3, and inhibits HRG1-b- and BTC-induced AKT signaling. Follow-up experiments showed effect on mouse tumor xenografts, and clinical trials are currently taking place.

Another use of models is to systematically perform *in silico* experiments by perturbating the network model with drugs (or combinations of them) to identify adrug combination that may produce a desired outcome (such as to block pro-growth pathways in cancer). Iadeviaia and colleagues developed and calibrated a model of Insulin-like growth factor 1 (IGF1) signaling [28].

Using the model, they predicted, and experimentally validated, that the combined inhibition of the MAPK and PI3K/AKT pathways optimally inhibited the signaling networks and decreased cell viability in a breast cancer cell line.

Biochemical models can also be used to elucidate the molecular mechanisms of action. Hendriks et al. [29] studied a p38 inhibitor postulated to block preferentially the phosphorylation of p38 on one substrate (MK2) versus another (ATF2). Combining detailed biochemical measurements of phospho-MK2 and phospho-ATF2 with a biochemical model, they found a stoichiometric effect in which excess of MK2 could lead to a complex MK2-inhibitor-p38 that would sequester p38, blocking the effect of p38 on ATF2 producing thus the opposite effect to the expected one.

### Logic-based models

Logic-based approaches have become a popular alternative to model signal transduction, since they are based on a simple formalism that can capture cause-effect relationships such as the effects of drug treatments [30-32].

Logic-based models can be used to describe the qualitative behavior of signaling networks even when a limited amount of experimental data is available. The simplest logic-based model is a Boolean model of a signaling pathway, where the phophorylation state of a species can be represented as an ON state (logical value of 1) to indicate the activation of the protein (kinase/phosphatase/transcription factor); analogously, an inactive protein can be represented by the OFF state (logical value of 0). The Boolean model of a signaling pathway can thus be thought of as a network where nodes represent proteins, which can either be on or off, and edges (or ‘hyperedges’, representing multiple edges between the same pair of nodes) represent logical operations between proteins (such as logic ANDs and ORs).

Because of their scalability, logic models can be trained against high-throuhgput experimental data, and thus generate large-scale cell-specific models. Differences between healthy versus diseased cell models point at potential targets for therapies: blocking an interaction that is only functional in a disease cell should have a disease-specific effect withouth affecting healthy cells. In a recent study [33], the authors trained a general protein signaling network to phosphoproteomic data generated in primary hepatocytes and four cell lines representing different stages of the development of hepatocellular carcinoma. A comparison of the resulting models revealed key differences between normal and transformed hepatocytes in three different pathways.

Furthermore, logic models can be used to understand drug’s mode of action, and in particular provide large-scale insights accross many different pathways, which cannot be modelled with biochemical models. In the study discussed above, the authors observed an effect of TPCA-1 (an IKK-inhibitor) on JAK/STAT signaling that could not be reconciled with their model based on prior knowledge of the involved pathways. Follow-up experiments including another IKK inhibitor (BMS345541) showed that the effect was specific to TPCA-1, and thus based on an off-target effect rather than an unknown crostalk between Ikb/NFkb and JAK2/STAT pathways [33]. In a related study [34], a similar procedure was used to generate models specific to certain drugs. By comparing the changes in the functional wiring with and without drugs, the authors found beside obvious effects (e.g., EGFR inhibitors blocks the EGFR pathway) not-reported alterations of signal transduction due to drug promiscuity (e.g. EGFR inhibitor Gefitinib inhibits IL1a-mediated activation of JNK activation).

Logic models can be used to systematically explore the properties of large networks to identify therapeutical targets. Saadatpour et al. performed a perturbation analysis of a model of signalling in T-cells, that lead to the identification of 19 potential targets for large granular lymphocyte leukemia, a diseased that exhibits an abnormal increase in the amount of T-cells [35].

In another application of logic modeling, Sahin et al. built a literature-derived logic model of ERBB receptor-regulated G1/S transition [36]. They used their model to investigate a chemotherapeutic resistant cell line (specifically, breast cancer cell with de novo tratuzumab resistance) and to find targets whose knockdown would increase drug senstivity. Furthermore, with their model the authors proposed (and experimentally validated in-vitro) c-MYC as a novel therapeutic target in both resistant and sensitive to trastuzumab breast cancer cell lines.

## Identifying drug mode of action: drug networks

The immediate interpretability of networks and the solid algorithmic background of graph-theory have been recently exploited in computational drug discovery, where “drug-networks” describing different kind of relationships among drugs, diseases, and molecular targets have been successfully developed. In what follows we review some of the most promising results achieved so far.

### Gene-Expression based networks for drug-classification and -repositioning

Differently from the networks described in the previous sections, a number of approaches has been developed on the idea of inferring drug-drug and/or drug-disease similarity networks from gene expression data. In drug networks, each node represents a drug (or disease) and each edge a significant similarity or “anti-similarity” between nodes. In order to build these networks, a number of issues must be solved. Most of them are linked to the integration of data coming from different experimental settings, which are often difficult to merge together without introducing biases, due to batch effects and individual-experiment specificities [37].

As shown in Figure 1C, the common hypothesis underlying these gene expression based methods is that every biological state can be described through a proper gene signature, which can be defined as a set of genes combined with a specific pattern of expression. By making use of “partial” or “genome-wide” metrics, similarity in gene expression signatures (or anti-similarity, when the sign of expression changes are reverted), summarising drug transcriptional responses and/or disease phenotypes, can be used to build drug-drug (DD) or drug-disease (Dd) networks. These networks, in turn, can be queried to identify the potential therapeutic effects and off-targets of a new molecule, by analysing the drugs connected to it in the network (“guilt-by-association” analysis), or to identify new applications for already approved drugs (i.e. “drug repositioning”). A comprehensive review on these two classes of methods has been recently published [38].

The usefulness of a Drug-drug network (Figure 1C) is based on the hypothesys that if gene signatures summarising the effect of drugs are significantly similar to each other, and hence the drugs are connected in the drug-drug network, then those drugs will likely share a common MoA [39].

Drug-disease networks rely on the following assumption: if the gene signature summarizing the effect of a drug is significantly anti-similar with the gene signature characterizing a disease state, then it is reasonable to hypothesise that the drug could “revert” the disease signature, hence the disease phenotype [40]

Based on these assumptions a significant number of new clinical applications for already existing drugs have been identified by querying drug-disease networks [41-44] or drug-drug networks [45,46].

To establish edges between drugs or between a drug and a disease in most of the approaches proposed so far, the authors used as reference dataset the Connectivity Map (cMap) [47]: the first large installment of gene expression profiles following drug treatment on five human cancer cell lines with more than 1,000 different bioactive small molecules. In this work, the authors also proposed a non parametric method based on the Kolmogorov-Smirnoff statistic [48] to find connections between a predefined gene signature and the gene expression profiles in the cMap [49].

Moving along these lines, Hu and Agarwal [50] inferred a drug-disease networks in which two nodes (that can represent both drugs and diseases) were connected if the corresponding signatures were significantly similar or significantly anti-similar. In order to compose disease signatures to be integrated with the cMap drug signatures, the authors mined the Gene Expression Omnibus (GEO) repository [51]. By analyzing the anti-similarities between drugs and disease in the network, they were able to predict new clinical applications for existing drugs, such as the potential efficacy of some antimalaria drugs in treating Chron’s disease, and that of other established drugs for Huntington disease.

While derived from similar principles, Iorio *et al* first generated a drug-drug network from the cMap dataset using a novel “drug distance metric” able to score the similarity between genome-wide GEPs following drug treatment [45]. The network was then analysed with graph-theoretic tools to identify “communities” of drugs consisting of groups of densely interconnected nodes (i.e. drugs). The authors found that these communities were indeed enriched for drugs sharing a common MoA and therapeutic application. The network was then used to classify both known and novel HSP90 inhibitors and CKD2 inhibitors by integrating them in the network and analysing the MoAs enriched in the surrounding communities. In addition, by analysing the neighborhood of a “seed compound” with a desired MoA they were able to predict and experimentally validate that Fasudil, a known ROCK inhibitor approved in Japan against blood vessel obstruction, may enhance cellular autophagy.

Taken together these results show the ability of drug networks in identifying novel applications for existing drugs, as well as to charachterise novel molecules by looking at the known properties of their connected neighboring compounds.

### Drug-Networks based on side-effects similarity

In addition to methods based on GEPs, other kinds of drug similarity metrics have been developed to infer drug networks.

Campillos *et al*[52] mined the Unified Medical Language System (UMLS) [53] ontology for medical symptoms and extracted informations about known side-effects of a large number of marketed drugs. They then assembled a network in which two drugs were connected by an edge if known to cause similar adverse reactions. In this way, they were able to discover unexpected connections among drugs with dissimilar chemical structure and different therapeutic indication. A number of these similarities were experimentally verified and confirmed previously unreported drug-target relations. Specifically, the authors predicted, and experimentally validated with in-vitro and cell assays, that the nervous system drugs pergolide, paroxetine, and fluoxetine share a common target with rabeprazole, a proton pump inhibitor, approved for relieving duodenal ulcer symptoms and for treating ulcerative gastroesophageal reflux disease.

## Conclusions

Molecular and drug networks are powerful approaches to speed up and improve both the drug discovery process and drug repositioning. We expect that their full integration into the academic and industrial drug discovery pipeline will likely have a major impact.

We have reviewed three different broad approaches used to identify drug MoA by harnessing the power of networks, at three different level of abstraction: transcriptional networks, protein networks and drug networks.

The role of transcriptional networks in determining mode of action of small molecules is undoubtebly growing, thanks to availability of more detailed gene network models and to the ever increasing amount and quality of GEPs. Indeed, thanks to the new sequencing technologies, it is now possible to achieve extreme multiplexing, i.e. mix different RNA samples in the same assay, thus enormously reducing the costs of obtaining GEPs in specific cell types and tissues. For example, a new high-throughput sequencing strategy was recently described [54], allowing measurement of gene expression profiles of hundreds of genes in thousands of samples to identify small molecules for therapy of hormone-refractory prostate cancer.

Signaling networks are the elective choice to model and identify drug Mode of Action, since most of the known effects of small molecules are mediated by kinases and phosphates. Despite the recent progress in fast and effective computational approaches, some of which were here reviewed, the major drawbacks is the experimental effort required to perform comprehensive phosphoproteomic time-course experiments. Increasing precision and decreasing costs of performing genome-wide phosphoproteomic measurements will likely lead to an increased use of signaling network models in the drug discovery.

Drug network are a unique tool to explore similarities and differences between drugs, and between drugs and diseases. The topology of “drug-drug” and “drug-disease” networks allows the inference of new applications for already approved drugs, and the elucidation of the MoA of small-molecules. Unlike transcriptional and signaling networks, these methods provide little or no mechanistic insights, since they rely on broad similarities in phenotypic effects of drugs, and not on detailed molecular interactions. Nevertheless, because of this, they do not require ad-hoc and expensive experiments, but can rely on the huge amount of publicly available transcriptional, chemoinformatics and literature-derived data, from which similarities and corresponding drug networks can be inferred. These data already exist and have not been fully exploited yet, and arguably it will be able to reveal a massive number of new meaningful connections.

Interestingly, at the time of writing, massive transcriptomics experiments are ongoing where thousands of cell lines are being treated with thousands of small molecules to detect gene expression changes following treatment with each compound (refer to the NIH LINCS initiative http://www.lincsproject.org). Such a trove of data can be analysed by network-based approaches to yield unprecedented insights connecting small molecules, pathways and diseases.

We have considered three different types of networks, each derived from different sources of experimental data or literature-based analysis. Each network type provides a different and potentially complementary view of the drug MoA with potential applications both in drug discovery/lead optimisation and for drug repositioning. It can be therefore expected that combining transcriptional, protein and drug networks will lead to an enhanced understanding of drug’s mode of action.

In order to achieve this long-term objective, however, some outstanding challenges should be solved. For example, it is not obvious how to predict from cell-based data, such as GEPs following treatment, the effect of a compund in a tissue or in the whole-organism. Moreover, the general applicability of these approaches in the face of cellular and/or patient heterogeneity has yet to be proven. Linking models of signalling to transcriptional networks would allow to integrate proteomics and gene-expression data, but how to best achieve this is still an open question [55]. Moreover, it is still not clear what a good drug should look like in context of networks, since no established criteria are available yet.

Ultimately, quantitative models of small molecule function promise to link molecular events at cellular level to phenotypical outcomes and to cellular and body-level effects.

## Competing interests

The authors declare that they have no competing interests.

## Authors’ contributions

All authors read and approved the final manuscript.
